# Supplementary material for: Free Cholesterol Accelerates Aβ Self-Assembly on Membranes at Physiological Concentration
Source: Int J Mol Sci. 2022 Mar 3;23(5):2803. doi: 10.3390/ijms23052803 (PMC8911190; doi:10.3390/ijms23052803)
Supplement: Supplementary file 1 [file ijms-23-02803-s001.zip › ijms-1613753-supplementary materials.pdf]

Free cholesterol accelerates Aβ self-assembly on membranes at physiological concentration

Mohtadin Hashemi[1,†], Siddhartha Banerjee[1,2, †], and Yuri L. Lyubchenko[1*]

[1]Department of Pharmaceutical Sciences, University of Nebraska Medical Center, 986025 Nebraska Medical Center, Omaha, NE 68198-6025

[2]Current affiliation: Department of Chemistry and Biochemistry, The University of Alabama, Shelby Hall, Tuscaloosa, AL 35487

[†]These authors contributed equally.

[*] Corresponding author e-mail: ylyubchenko@unmc.edu

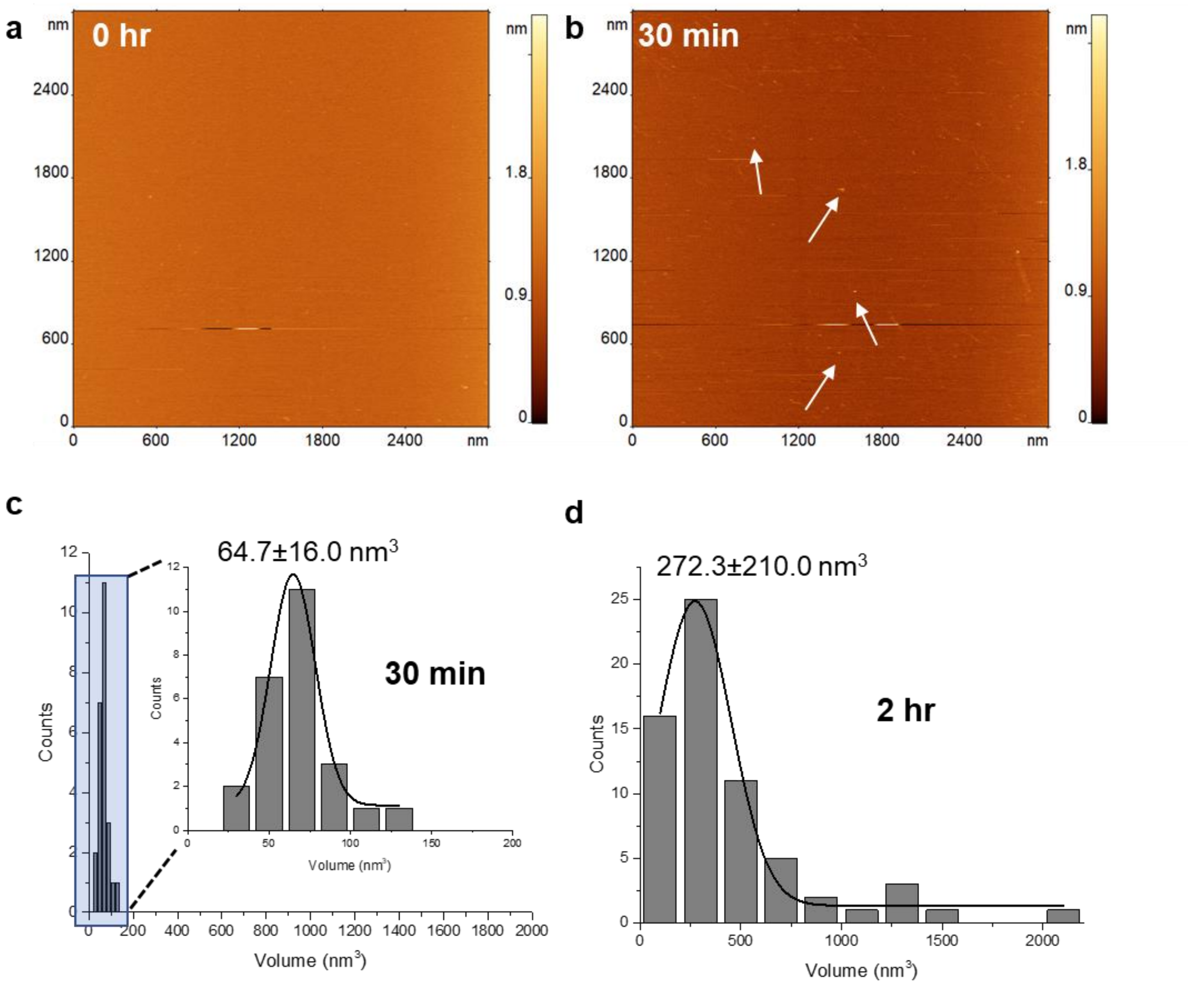

Figure S1. Aggregation of 10 nM Aβ(1-42), in the presence of 100 nM Chol, on PC-PS-Chol bilayer. **A)** AFM image of PC-PS-Chol lipid bilayer before addition of Aβ(1-42)-Chol solution. The smooth, homogeneous bilayer surface is ideal for monitoring the aggregation events. **B)** The same area of the surface after 30 min following the addition of Aβ(1-42)-Chol solution. Small aggregates are visible on surface (highlighted with white arrows). The distribution of aggregate volumes after 30 min, **c)**, and 2 hr, **d)**. Peak values obtained using Gaussian fits are presented in the histograms; errors indicate standard deviation.

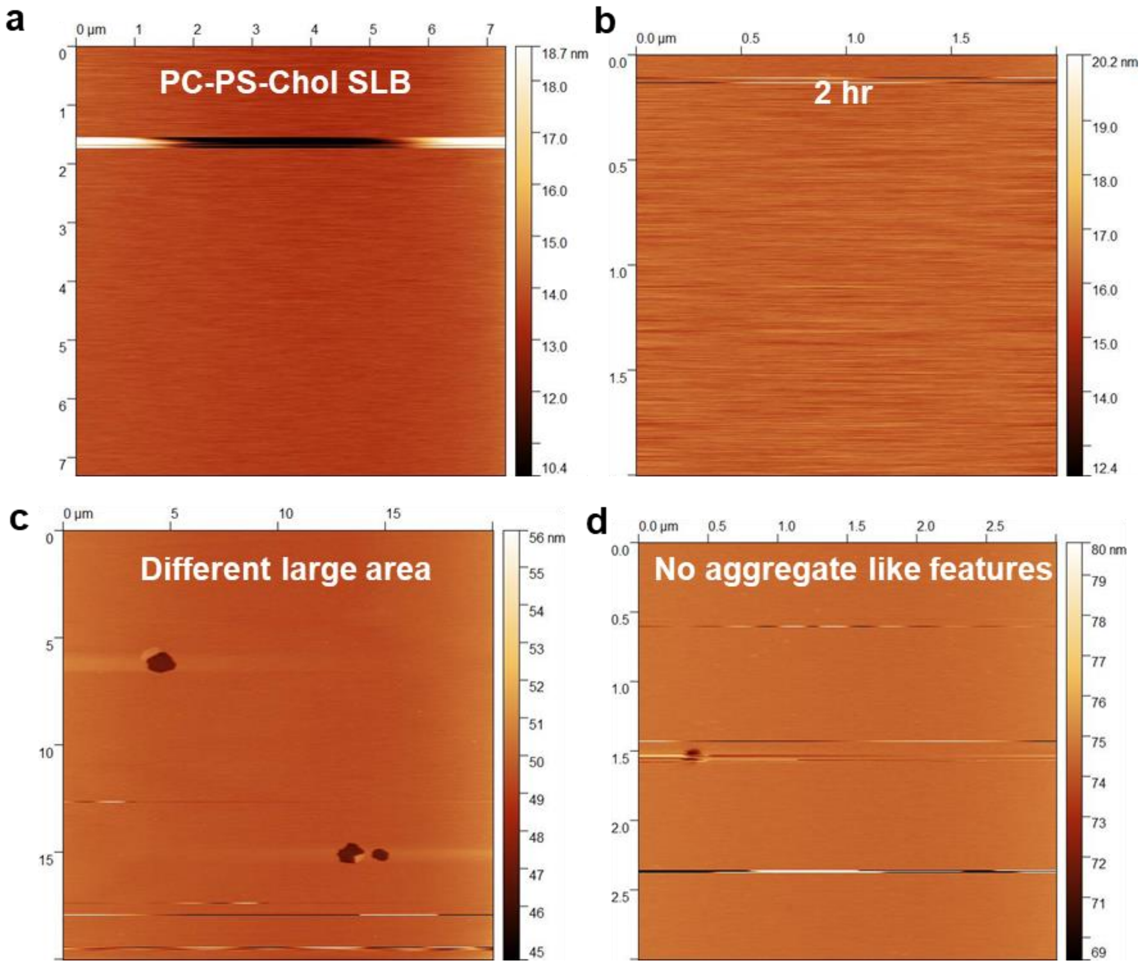

Figure S2. Time-lapse images of PC-PS-Chol bilayer in presence of 100 nM Chol. **a)** The PC-PS-Chol bilayer surface before addition of Chol solution. **b)** Bilayer surface 2 hr after addition of Chol solution. No aggregate-like features are observed. **c)** A large-scale image of a different area of the bilayer surface after 2 hr incubation with Chol solution. d) Zoom of **c** to check for aggregate-like features.

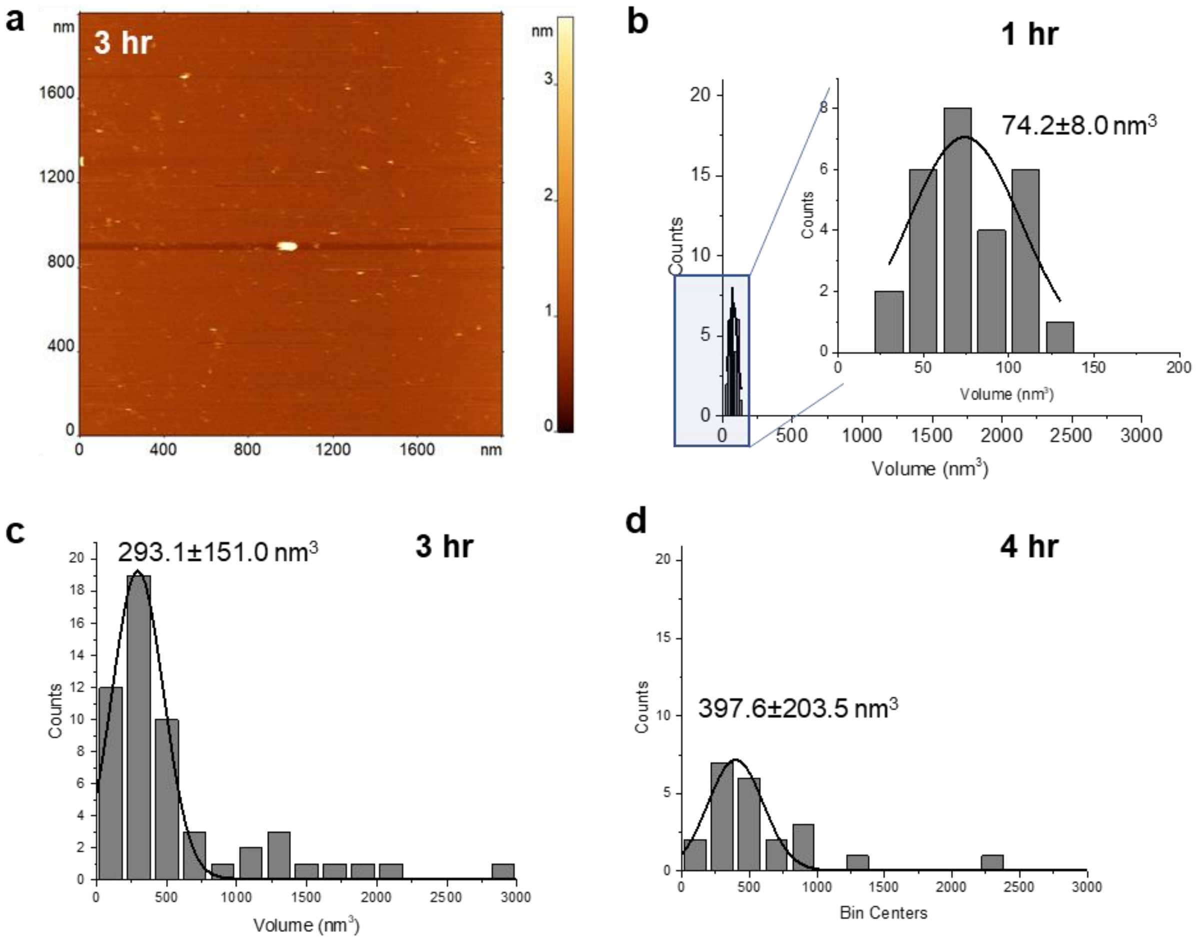

Figure S3. Dynamics of Aβ(1-42) aggregation on the PC-PS-Chol bilayer. **a)** AFM image of the bilayer surface recorded 3 hr after addition of Aβ(1-42)-Chol solution. **b-d)** Bar diagrams showing the distribution of aggregate volumes at different incubation time points.

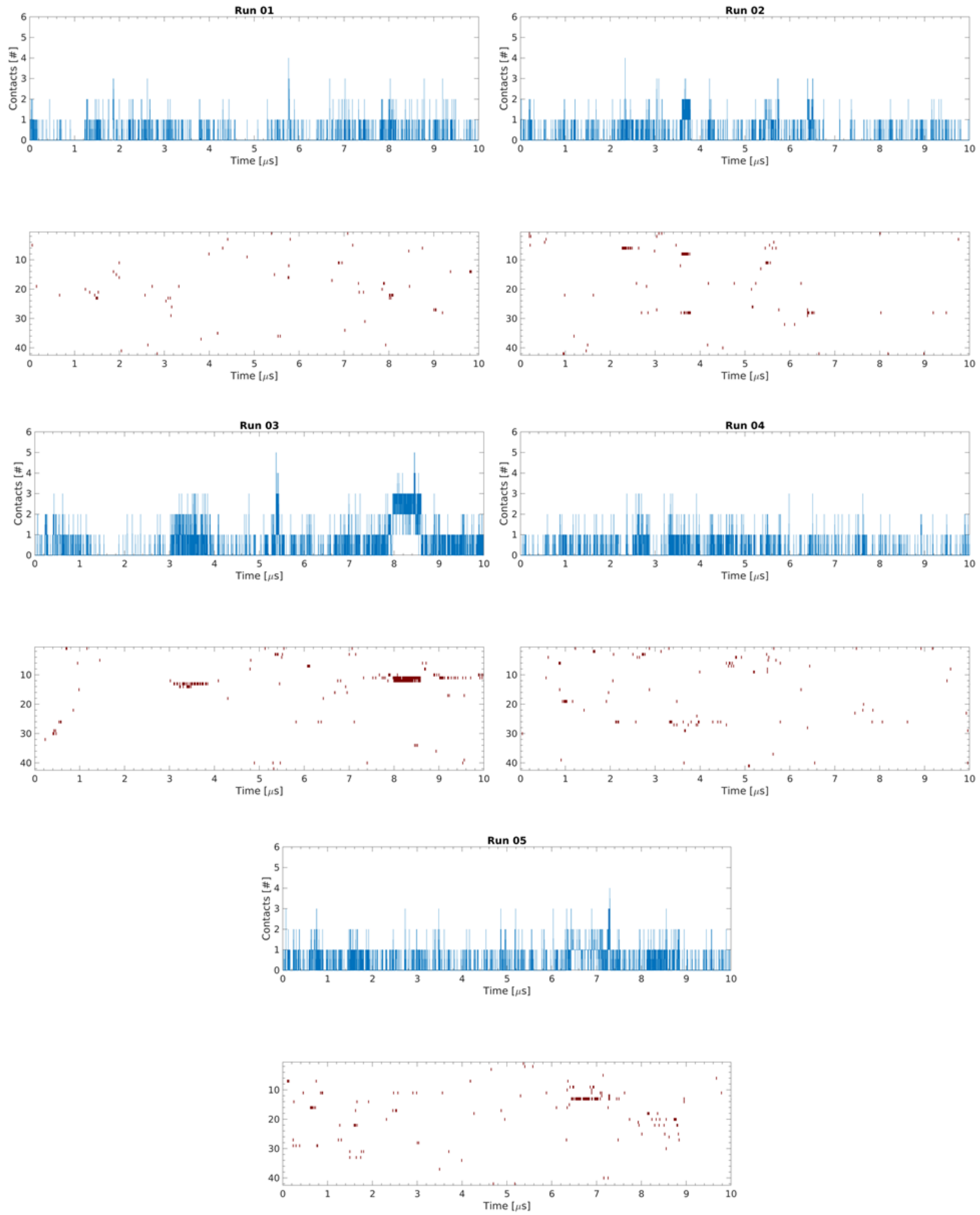

Figure S4. Aβ(1-42)-Chol contacts. Evolution of contacts between Chol molecule and residues of Aβ(1-42) monomer for each simulation. Blue graphs show cumulative contacts while the red kymographs show which Aβ(1-42) residues are responsible for contact.

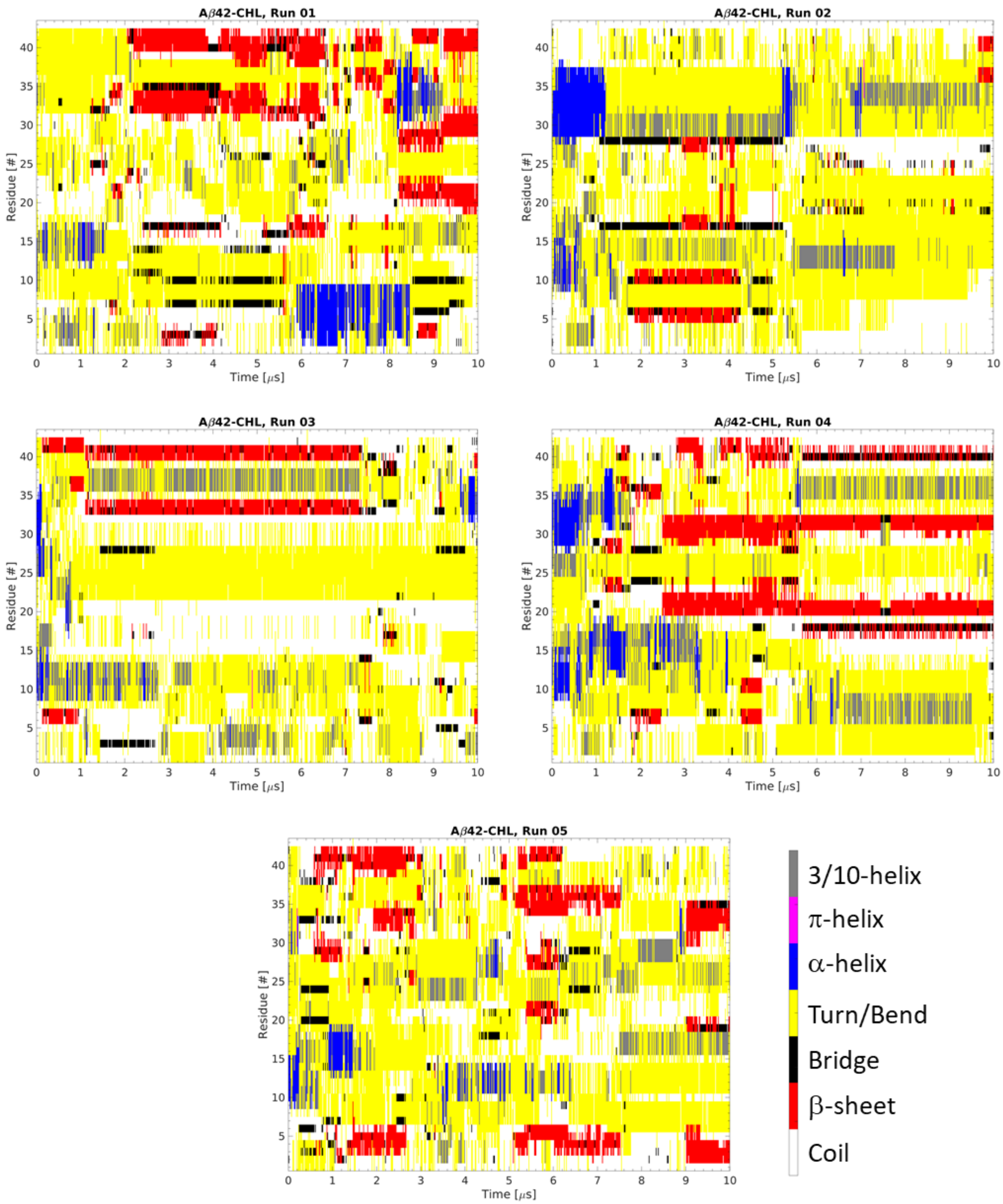

Figure S5. Secondary structure maps for Aβ(1-42) monomer interacting with Chol. Each map represents one simulation.

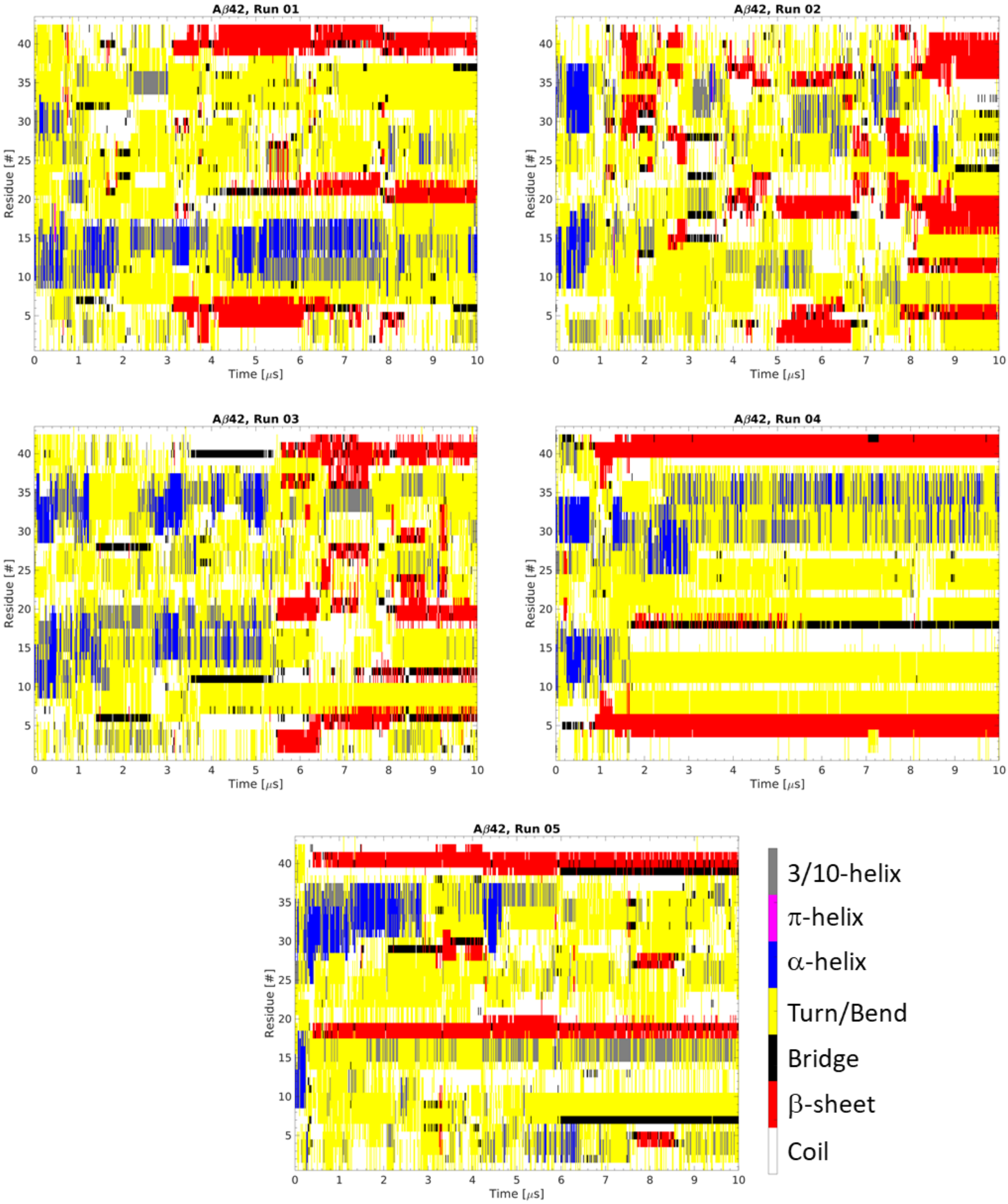

Figure S6. Secondary structure maps for Aβ(1-42) monomer. Each map represents one simulation.
